# Monitoring tar spot disease in corn at different canopy and temporal levels using aerial multispectral imaging and machine learning

**DOI:** 10.3389/fpls.2022.1077403

**Published:** 2023-01-23

**Authors:** Chongyuan Zhang, Brenden Lane, Mariela Fernández-Campos, Andres Cruz-Sancan, Da-Young Lee, Carlos Gongora-Canul, Tiffanna J. Ross, Camila R. Da Silva, Darcy E. P. Telenko, Stephen B. Goodwin, Steven R. Scofield, Sungchan Oh, Jinha Jung, C. D. Cruz

**Affiliations:** ^1^ Department of Botany and Plant Pathology, Purdue University, West Lafayette, IN, United States; ^2^ Tecnológico Nacional de México, Instituto Tecnológico de Conkal, Yucatán, Mexico; ^3^ USDA-Agricultural Research Service, Crop Production and Pest Control Research Unit, West Lafayette, IN, United States; ^4^ Institute for Plant Sciences, Purdue University, West Lafayette, IN, United States; ^5^ Lyles School of Civil Engineering, Purdue University, West Lafayette, IN, United States

**Keywords:** maize, disease modeling, epidemics, fungus, phyllachora maydis, plant disease, unmanned aircraft systems

## Abstract

**Introduction:**

Tar spot is a high-profile disease, causing various degrees of yield losses on corn (*Zea mays* L.) in several countries throughout the Americas. Disease symptoms usually appear at the lower canopy in corn fields with a history of tar spot infection, making it difficult to monitor the disease with unmanned aircraft systems (UAS) because of occlusion.

**Methods:**

UAS-based multispectral imaging and machine learning were used to monitor tar spot at different canopy and temporal levels and extract epidemiological parameters from multiple treatments. Disease severity was assessed visually at three canopy levels within micro-plots, while aerial images were gathered by UASs equipped with multispectral cameras. Both disease severity and multispectral images were collected from five to eleven time points each year for two years. Image-based features, such as single-band reflectance, vegetation indices (VIs), and their statistics, were extracted from ortho-mosaic images and used as inputs for machine learning to develop disease quantification models.

**Results and discussion:**

The developed models showed encouraging performance in estimating disease severity at different canopy levels in both years (coefficient of determination up to 0.93 and Lin’s concordance correlation coefficient up to 0.97). Epidemiological parameters, including initial disease severity or y_0_ and area under the disease progress curve, were modeled using data derived from multispectral imaging. In addition, results illustrated that digital phenotyping technologies could be used to monitor the onset of tar spot when disease severity is relatively low (< 1%) and evaluate the efficacy of disease management tactics under micro-plot conditions. Further studies are required to apply and validate our methods to large corn fields.

## 1 Introduction

Tar spot, caused by *Phyllachora maydis* Maubl., is a relatively new and high-profile disease in the United States (Ruhl et al., 2016). Since the early-to-mid 1900s, tar spot has been prevalent in Mexico, the Caribbean, and Central and South America (Valle-Torres et al., 2020). The fungus *P. maydis* is a presumably obligate parasite that forms black stromata, which are small, raised, round to semi-circular masses of fungal tissue that contain spore-bearing structures. In some cases, tan or brown lesions appear around the stromata, forming ‘fisheye’ lesions (Bajet et al., 1994; Kleczewski et al., 2019; Valle-Torres et al., 2020). *P. maydis* can infect corn leaves, leaf sheaths, and husks, cause premature senescence and reduce grain and forage yield and quality (Bajet et al., 1994; Kleczewski et al., 2019; Telenko et al., 2019). It is reported that tar spot can reduce the grain yield of susceptible hybrids by up to 2,900 kg/ha (58%) in Mexico (Loladze et al., 2019). During the 2018 epidemic in the Midwestern United States, tar spot caused minor to severe infection (40 – 50% leaf surface with symptoms) and yield loss of up to 4,035 kg/ha in hybrids during corn performance trials (Telenko et al., 2019). In 2021, tar spot caused a grain yield loss of 5.88 million metric tons with an economic impact of US$1.25 billion for the United States, with a 1.44% decline in grain yield (Crop Protection Network, 2022).

Plant disease evaluation, including identification and quantification, is traditionally conducted by highly trained personnel for crop production or research purposes. However, visual assessment of diseases is susceptible to subjectivity and errors introduced by human raters, such as variation in ability, value preferences, lesion number and size relative to the area infected, the complexity of symptoms and timing (Bock et al., 2010). In addition, it is also time-consuming and costly to train personnel and improve visual assessment accuracy. Digital phenotyping technologies offer an opportunity to enhance the objectivity and efficiency of plant disease detection and quantification (Lee et al., 2021).

Digital phenotyping technologies, such as automation, sensing and photography, and computer vision, have been evaluated and used to monitor plant diseases, including data acquisition, mining, and analysis (Mahlein, 2016; Bock et al., 2020). Various platforms such as smartphones, robots, airplanes, unmanned aircraft systems (UASs), and satellites have been used to acquire data for plant disease detection (Nilsson, 1995; Qin and Zhang, 2005; Franke and Menz, 2007; Johannes et al., 2017; Chen et al., 2018; Barman et al., 2020; Cubero et al., 2020; Tetila et al., 2020; Rodríguez et al., 2021). Among these platforms, UASs are of interest to researchers and producers due to their high throughput in data acquisition, the flexibility of deployment, and relatively low cost (compared to imaging methods based on robots, airplanes, and satellites). Along with these platforms, different sensors and imagers, such as red-green-blue or RGB, multispectral, hyperspectral, and thermal cameras, are deployed to collect data (Zhang et al., 2019a; Deng et al., 2020; Tetila et al., 2020). Vegetation indices (VIs), texture, thermal, and morphological features (e.g., canopy cover and volume and contour) are extracted from data for plant disease monitoring (Zhang et al., 2019a; Lee et al., 2021; Vishnoi et al., 2021). Machine learning algorithms are commonly applied to data collected or features extracted to automatically identify, classify, and quantify plant diseases (Johannes et al., 2017; Wang et al., 2020; Fernández-Campos et al., 2021; Gao et al., 2021).

UAS-based remote sensing, along with machine learning, has been applied to plant disease monitoring, demonstrating encouraging results in many studies (Huang et al., 2019; Stewart et al., 2019; Zhang et al., 2019a; Zhang et al., 2019c; Chivasa et al., 2020; Wang et al., 2020). For instance, UAS-based multispectral imaging was used to monitor the resistance of twenty-five corn varieties to maize streak virus (Chivasa et al., 2020). Significant correlations between data derived from UAS and visual scores were observed in the study (r = 0.74 – 0.84); through random forest classifiers and optimized variables, corn varieties were classified into three resistance levels to maize streak virus with overall classification accuracies of 77.3% and Kappa of 0.64. In addition, many studies attempted to map the spatial or horizontal distribution of plant disease using UAS-based remote sensing (Smigaj et al., 2015; Deng et al., 2020; Wang et al., 2020; Ye et al., 2020). Wang et al. evaluated UAS-based remote sensing and different machine learning classifiers (supervised, unsupervised, and combined unsupervised classification) to automatically map cotton root rot-infested fields (Wang et al., 2020). One of their proposed new methods that utilized k-means segmentation and morphological information outperformed other methods in classifying healthy and diseased plants with an overall accuracy of 88.5%. In contrast to these studies, where disease detection and mapping focused on horizontal distribution over the top canopy, research that monitors disease distribution in vertical space (from lower to upper canopy) using remote sensing has not been well studied. Oh et al. quantified tar spot intensity at the middle and upper canopy using UAS-based remote sensing; however, the lower canopy was not included in the study (Oh et al., 2021). Developing methods to monitor plant diseases at various canopy levels is critical for epidemiological modeling and disease management (Gongora-Canul et al., 2020).

Different regression models have delineated temporal dynamics of epidemics in which disease progress and crop responses were monitored (e.g., disease resistance and yield loss) (Berger, 1981; Madden et al., 2000; Jeger and Viljanen-Rollinson, 2001; Gongora-Canul et al., 2020). Population-dynamics models, including exponential, logistic, monomolecular, and Gompertz models, are valuable for representing, comparing, and understanding plant disease epidemics (Gongora-Canul et al., 2020). Meanwhile, spectral data acquired by sensors from multiple time points have been used for disease monitoring in many studies; however, spectral data from multiple time points were analyzed separately, not temporally, in most cases (Franke and Menz, 2007; Zhang et al., 2019a; Gongora-Canul et al., 2020). Previous studies using UAS-based remote sensing have not characterized the temporal development of diseases using population-dynamics models and associated epidemiological parameters. Research to characterize the temporal dynamics of a disease with UAS-based spectral data is desired and could facilitate disease surveillance and on-time management.

Efficient and scalable phenotyping technologies, for example, integration of UAS-based imaging and machine learning, are needed for detecting and quantifying the vertical distribution and temporal development of plant diseases. Such phenotyping information is crucial to crop disease modeling and management (Gongora-Canul et al., 2020). In addition, Practical applications of digital phenotyping technologies in disease monitoring interest various stakeholders, including growers, agricultural consultants, and researchers (Zhang et al., 2019b). We hypothesized that digital phenotyping technologies using UAS-based multispectral imaging and machine learning offer an opportunity to detect and quantify the vertical distribution and temporal development of plant diseases. To test the hypothesis, we used tar spot of corn as a model system and focused on the following objectives: (1) estimate the vertical distribution (lower, middle, and upper canopy) of tar spot disease within the corn canopy with UAS-based multispectral imaging and machine learning; (2) monitor the temporal development of tar spot using data derived from multispectral imaging; and (3) evaluate the application of information derived from digital phenotyping technologies on disease monitoring (e.g., aerial detection of the onset of tar spot and comparison of disease management tactics).

## 2 Materials and methods

### 2.1 Tar spot experiment setup

Field experiments were conducted in 2020 and 2021 at the Pinney Purdue Agriculture Center (PPAC, 41°27’20.13”N, 86°56’28.58”W), Indiana, United States, a location known for its high risk for tar spot development (Telenko and Creswell, 2019). Four and two experiments were involved in 2020 and 2021, respectively (**Supplementary Figures 1, 2**). Different combinations of hybrids, tillage types, fungicides, and fungicide application timing were evaluated in various experiments, creating variations in disease severity (**Table 1**). All experiments were organized in a randomized complete block design with four replications. Each micro-plot in the experiments consisted of four rows of corn with 76 cm row spacing and 10 m or 9 m micro-plot length (84,000 plants per ha). Plants relied on natural infection caused by the presence of *P. maydis* in the environment.

**Table 1:** Details of tar spot experiments during the 2020 and 2021 growing seasons.

Year	Trial name	Effects studied	Planting date	Number of treatments	Number of Hybrid(s)	Tillage type	Number of fungicide treatments	Number of data acquisition
2020	Tar 4^a^	Fungicide timing	8-Jun-20	10	1	Strip	9 + 1 control	9
	Tar 3^b^	Fungicide efficacy and timing	9-Jun-20	18	1	Strip	16 + 2 controls	11
	Tar 1^c^	Fungicide efficacy	9-Jun-20	10	1	Strip	9 + 1 control	10
	Tar 2^d^	Tillage, hybrids, and fungicide	6-Jun-20	12	3	Strip, Conventional	1 + 1 control	10
2021	Tar 2^d^	Tillage, hybrids, and fungicide	26-May-21	12	3	Strip, Conventional	1 + 1 control	9
	Tar 3^e^	Fungicide efficacy and timing	27-May-21	18	1	Strip	16 + 2 controls	5

a (Ross et al., 2021b).

b (Da Silva et al., 2021).

c (Ross et al., 2021a).

d Ross et al., personal communication.

e (Da Silva et al., 2022).

### 2.2 Visual assessment of disease severity and UAS-based multispectral imaging

Data acquisition through visual assessment and UAS-based multispectral imaging was carried out weekly or biweekly by two groups of trained plant pathologists in two years, and the time of data acquisition was recorded as day after planting or DAP. Visual evaluation of disease severity consisted of quantifying the ratio of leaf area with stromata or chlorosis/necrosis to total leaf area multiplied by 100 (Oh et al., 2021). Disease severity of tar spot in 2020 was evaluated by the first group of pathologists based on the combination of stromata or chlorosis/necrosis (or short for Chl/Nec) symptoms. However, the severity of stromata and Chl/Nec in 2021 were evaluated separately by the second group of pathologists. Visual phenotyping standards often differ among laboratories, and for that reason, visual assessment data were modified to bring the disease severity of 2021 to a scale that did not exceed 100% severity per leaf. Hence, when the severity based on stromata was greater than that based on Chl/Nec, severity based on stromata was used, and vice versa. The reason for this transformation is that at the early stage of disease development, stromata were the prevalent symptom, while towards the end of the growing season, Chl/Nec was more dominant. Disease severity was evaluated on one leaf from the lower, middle and upper canopies. In this study, the ear leaf was considered as L0, while leaves below or above the ear leaf were labeled as L- and L+ (plus a number), respectively. Therefore, a corn plant’s given lower, middle, and upper canopy included leaves from L - n (lowest leaf) to L - 3, L - 2 to L + 1, and L + 2 to L + n (flag leaf), respectively. The middle two rows in each micro-plot were used in the visual assessment to avoid potential treatment overlaps from neighboring micro-plots, and assessment was conducted from around the tasseling (VT) to physiological maturity (R6) stages for all trials and both years (Oh et al., 2021).

Images were acquired by a multispectral camera mounted on a Phantom 4 Multispectral UAS (SZ DJI Technology Co., Ltd., Shenzen, China) in 2020 and a MicaSense Rededge-M camera (MicaSense Inc., Seattle, WA, USA) mounted on a Matrice 200 UAS (SZ DJI Technology Co., Ltd., Shenzen, China) in 2021. Due to the differences in cameras, each year was considered an independent environment and no model transferring was conducted. Before aerial image acquisition, images of a reflectance panel (MicaSense Inc., Seattle, WA, USA) were acquired at ground level for radiometric calibration. UASs were programmed to collect data at altitudes of 30 m in 2020 and 50 m in 2021 with front and side overlaps of 75% and flight speed of 3 m per second, which resulted in ground sampling distances (GSD) of ~1.5 and ~3.5 cm for orthomosaic images derived from the cameras as mentioned, respectively. Considering weather conditions that limited UAS operations at given times, images were acquired within two days of the dates for visual assessment with one exception (within four days).

### 2.3 Image processing and feature extraction

**Figure 1:**
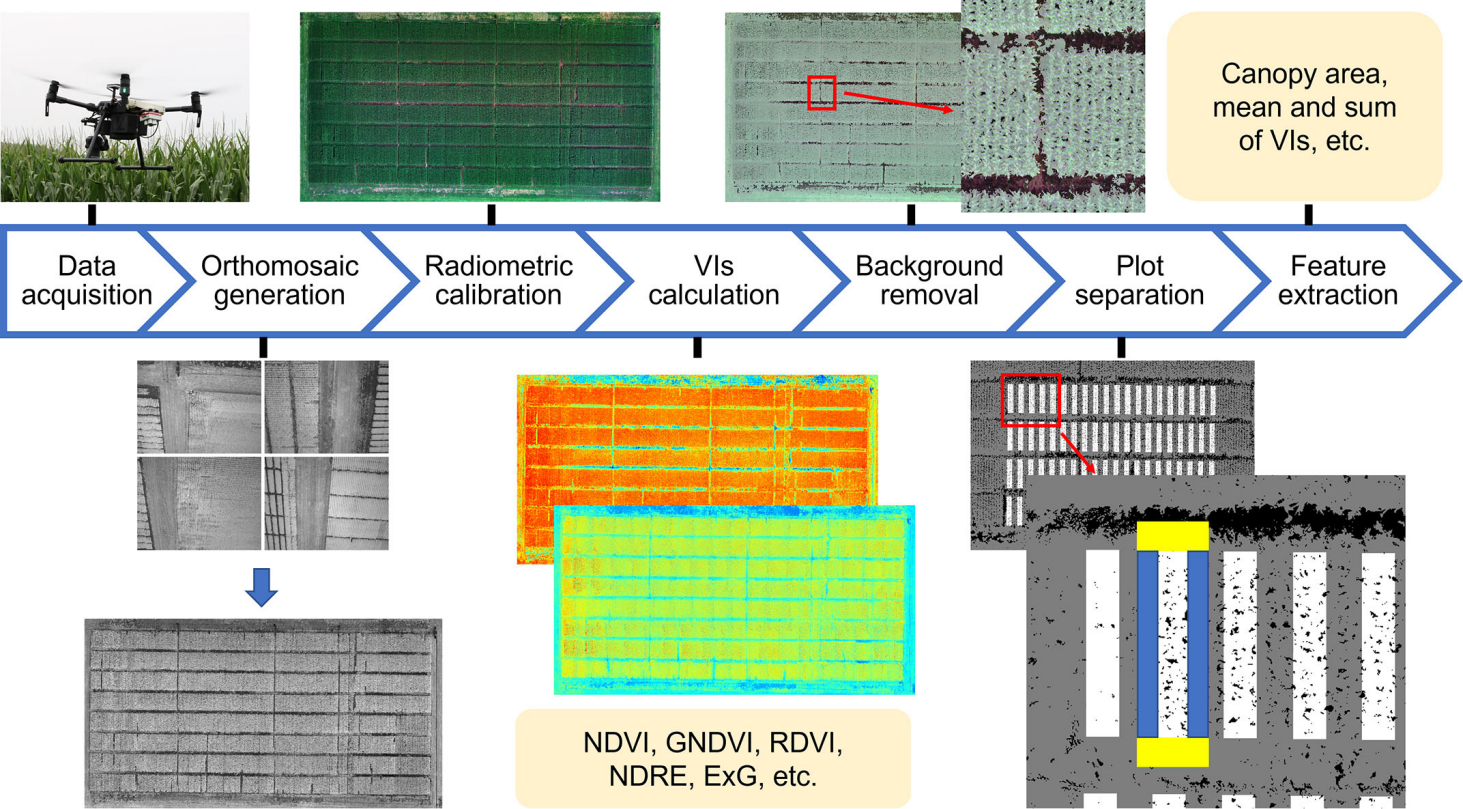
The procedure of image processing and feature extraction. VIs, vegetation indices; NDVI, normalized difference vegetation index; GNDVI, green normalized difference vegetation index; RDVI, renormalized difference vegetation index; NDRE, red edge normalized difference vegetation index; ExG, excess green; blue and yellow rectangles in micro-plot separation step indicate rows and beginning and end of rows of micro-plots that were excluded from multispectral information extraction.

Individual multispectral images obtained were processed in Metashape (AgiSoft LLC, St. Petersburg, Russia) and DroneDeploy (DroneDeploy, San Francisco, California, USA) to generate ortho-mosaic images covering the entire experiment(s), as shown in **Figure 1**. Images were radiometrically calibrated with irradiance obtained by a downwelling light sensor and reflectance panel. Orthomosaic images were then processed with a customized pipeline developed in MATLAB (MathWorks Inc., 2021). Vegetation indices (VIs), such as normalized difference vegetation index (NDVI), simple ratio (SR), renormalized difference vegetation index (RDVI), among others, were calculated from different combinations of spectral bands for feature extraction in a later step (Zarco-Tejada et al., 2005; Harris Geospatial Solutions, 2022). More details about VIs used in this study can be found in **Supplementary Table 1**, and RDVI and NDRE maps for plots from different DAP of the Tar 2 trial of 2021 can be found in **Supplementary Figures 3, 4**. Before feature extraction, backgrounds such as soil and shaded canopy, in the ortho-mosaic images were removed to minimize their influence on VIs. For instance, the shaded canopy can lead to higher values for NDVI and SR while lower values for RDVI, compared with a sunlit canopy (Zhang et al., 2015). For that reason, we used the reflectance from upper and middle canopies to estimate the disease severity of the lower canopy. Pixels were considered as background and removed if the ‘value’ (a relative intensity of light) in the hue, saturation, and value (HSV) color space and RDVI value was less than thresholds (0.05 – 0.25 and 0.13 for ‘value’ and 2.00 – 2.50 and 0 for RDVI in 2020 and 2021, respectively). Thresholds for ‘value’ and RDVI were selected and adjusted based on visual inspection of sunlit and shaded canopy, vegetation, soil, and quality of foreground after background removal. Following that, micro-plots (3 m by 9 or 10 m) in each experiment were separated semi-automatically by a customized pipeline, where the user only needs to identify four corners of the experiment field in the ortho-mosaic image and the pipeline extrapolates the coordinates of each micro-plot. More details about the usage of the pipeline can be found in Zhang et al. (2019a). Corresponding to the visual assessment procedure, the multispectral information in the middle two planting rows was used for feature extraction. Edges at the beginning and end of the two rows of micro-plots were excluded to avoid border effects (**Figure 1**). With the four corners of each micro-plot, image-based features including canopy area, green canopy area, and mean, median, sum, and standard deviation values of five spectral bands and VIs were extracted.

### 2.4 Modeling of disease severity estimation and disease progression

Disease severity estimation models were developed using features extracted along with visual assessment. The features from multispectral images were normalized using equation (1) to avoid the impact of differences in data scales, for example, NDVI ranges from -1 to 1, while RDVI varies from -15 to 15.


(1) $$\hat{F}=\left(F-mean\right)/std$$

Where $\hat{F}$ is the normalized value of a feature for a micro-plot, *F* is the raw value of a feature for a micro-plot, while mean and std are the average and standard deviation of a feature. Least absolute shrinkage and selection operator (LASSO) regression was used to develop models to estimate disease severity at different canopy levels. LASSO develops linear regression models by removing redundant predictors (or features in our case) and fitting least-squares regression coefficients between predictors and response (or disease severity in our case). Normalized features were partitioned into training and testing datasets at a ratio of 3:1, and stratification partition was implemented based on the date of data collection. Models were developed and tested with training and testing datasets along with disease severity from different canopy levels, and specifications of parameters used in LASSO can be found in **Table 2**. The whole procedure of model development and testing was repeated four times (four iterations) with resampling of training and testing data. Estimated disease severity (from the developed model) was compared with actual disease severity (assessed by human raters). Performance of disease estimation models was evaluated by coefficient of determination (R^2^, measuring the precision between estimated and actual disease severity), root mean square error (RMSE), and Lin’s Concordance Correlation Coefficient (Lin’s CCC, measuring the accuracy that is a product of precision and bias, shown in equation 2) (Madden et al., 2007).

**Table 2:** Specifications of least absolute shrinkage and selection operator regression (LASSO).

Parameter	Value/Method
Ratio of training and testing data	3:1
Data partitioning for stratification	Partitioned by data collection dates
Weight of lasso versus ridge optimization (α)	1
Number of cross validations	5
Number of Monte Carlo repetitions for cross validation	3
Predictor selection method within cross-validated models	Minimum mean square error
Number of repeated five-fold cross validation	4


(2) $${\text{Lin's CCC or ρ}}_{\text{c}}=\frac{2*\sigma_{uw}}{{\left(\mu_{u}-\mu_{w}\right)}^{2}+\sigma_{u}^{2}+\sigma_{w}^{2}}$$

Where *μ*_u_ and *μ*_w_ are means from estimated and actual disease severity, $\sigma_{u}^{2}$ and $\sigma_{w}^{2}$ are variances, and *σ_uw_* is the covariances calculated from estimated and actual disease severity. In addition, transferability of estimation models between trials was evaluated with data collected in 2021. For example, models (or coefficients for linear regression) developed with multispectral features from the Tar 2 trial were used to estimate the visual assessment of the Tar 3 trial with the help of multispectral features from the Tar 3 trial, and vice versa.

Temporal development or disease progress of tar spot were modeled using actual and estimated disease severity to evaluate the feasibility of monitoring disease progress through multispectral imaging. The logistic regression curve, a model that has previously been shown to fit tar spot severity data (Gongora-Canul et al., unpublished), was used to depict disease progress empirically; the equation (3) used for modeling is shown below (Neher and Campbell, 1992).


(3) $$y=K_{max}/\left[1+\text{exp}\left(-\ln\left(\frac{y_{0}}{K_{max}-y_{0}}\right)-r_{L}*t\right)\right]$$

Where y is the disease severity at the time of data collection, y_0_ is the initial disease severity, K_max_ is the asymptote or the maximum level of disease intensity, r_L_ is the apparent infection rate, t is the time of data collection. The logistic disease progress curves were modeled using the nonlinear least-squares solver (lsqcurvefit function in MATLAB, version R2021a) and fitted independently for visual and the estimated disease severities according to different treatments (Coleman and Li, 1994; Coleman and Li, 1996). Key parameters depicting disease progress, including y_0_, K_max_, r_L_, and area under the disease progress curve (AUDPC), were modeled during curve fitting. K_max_ was modeled, instead of assuming 100% as the maximum disease severity, to avoid underestimation of the disease growth rate (Neher and Campbell, 1992). Parameters extracted from curves modeled using actual and estimated disease severity were compared using t-tests in SAS (SAS Institute, Cary, NC, United States). The comparison of parameters was conducted by year and canopy level.

### 2.5 Application of digital phenotyping technologies in disease monitoring

UAS-based detection of tar spot onset and quantification can be less reliable compared to the field-based visual method, because an individual stroma (0.5 – 2.5 mm wide by 2 – 3 mm long) cannot be delineated from UAS images taken for a regular mapping mission of an agricultural field (GSD of 3.5 cm for multispectral images collected at 50 m in this study) (Rocco da Silva et al., 2021). Therefore, we performed tar spot detection for each trial (**Table 1**) using UAS-based imaging with a slackened voting approach as shown below. From this approach, tar spot was considered present in a trial when at least a specific percentage of micro-plots was found to be infected by UAS-based disease detection.

A detailed procedure for the slackened voting approach is as follows. First, the best model for each canopy level was selected from four cross-validated models to estimate disease severity of each micro-plot. Second, the number of micro-plots with estimated disease severity greater than zero was counted. The number of micro-plots with tar spot for different combinations of trials and the three canopy levels was converted to a percentage, resulting in the estimated incidence rate per data collection date. Third, different thresholds, such as 5%, 10%, 20%, 30%, 40%, and 50% of micro-plots, were tested to determine if tar spot presented in a trial and the false warning rates (or the number of false negatives and positives of tar spot onset to the number of micro-plots) were calculated to select the best threshold. For example, when 10% of micro-plots with estimated disease severity over zero was used to determine if tar spot presented in the trial, it resulted in a false warning rate of 4%. Fourth, actual incidence rate and maximum actual disease severity for different combinations of trials, DAP, and canopy levels were obtained as ground references to evaluate the performance of onset detection. Sensitivity of detection was computed by dividing the number of infected plots identified correctly by estimated disease severity to the number of actual infected plots.

The efficacy of disease management tactics evaluated by digital phenotyping was compared to that of visual assessment. Spearman’s rank correlation (*r*_s_) was used to evaluate the similarity of rankings of treatments assessed by digital phenotyping and visual assessment. Spearman correlation analysis was applied to each trial for two years, and data from each time point and canopy level were also analyzed independently.

## 3 Results

### 3.1 Estimation of disease severity at different canopy levels

The actual disease severity (assessed by human raters) generally increased from the lower to the upper canopy at each time point of data collection and correlations of disease severities between canopy levels were observed. For example, based on actual disease severity data collected in 2020, the Pearson correlation coefficients were 0.88 between lower and middle canopy, and 0.73 between lower and upper canopies. Such correlations of disease severities between canopy levels were the foundation of estimating disease severity at different canopy levels using UAS-based multispectral imaging.

**Figure 2:**
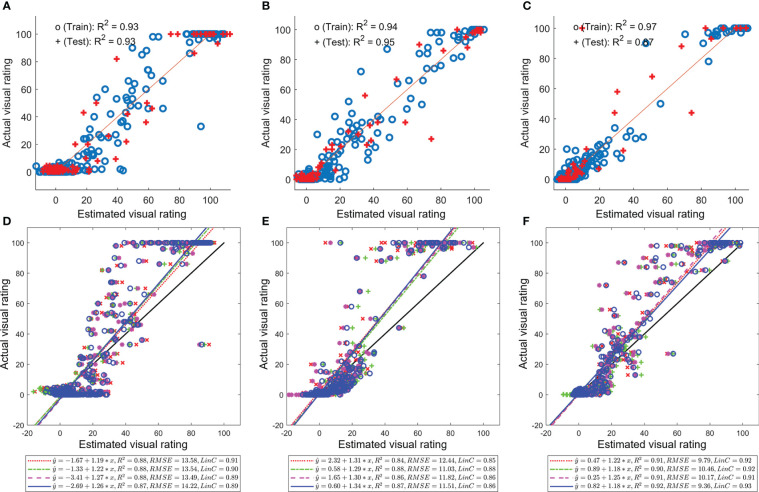
Estimation of visual assessment using image-based features and machine learning. **(A–C)** models trained and tested with data from the Tar 3 trial in 2021 (results of one iteration of training and testing); **(D–F)** evaluation of model transferability using parameters from models of the Tar 2 trial and data from the Tar 3 trial; **(A, D)** results of estimating visual assessment for the lower canopy; **(B, E)** results for the middle canopy; **(C, F)** results for the upper canopy.

Models developed with UAS-based multispectral imaging and machine learning demonstrated encouraging performance in estimating disease severity during both years and three canopy levels (**Table 3**). Estimation precision (coefficients of determination or R^2^) of models for training and testing data was greater than 0.75 in most cases, and the highest testing precision reached 0.93; RMSEs were less than 12% in all cases. Moderate to high agreements (Lin’s CCC of 0.75 to 0.97) between estimated and actual disease severity were also observed. At the same time, coefficients and intercepts or biases for linear relations between estimated and actual visual assessment were very close to one and zero, respectively. Models for the Tar 3 trial in 2021 demonstrated better performance with high estimation precision (over 0.91) and Lin’s CCC (over 0.96), regardless of canopy levels (**Figures 2A–C**). Image-based features, for instance, green canopy area, the mean of blue band, the median of MCARI2, the median of RDVI, the sum of red band, and standard deviation from most of the five spectral bands and VIs were frequently selected as predictors. Meanwhile, features selected by LASSO varied between years or canopies, and models for 2020 used more features (49 – 58 features) than those for 2021 (20 – 34 feature).

**Table 3:** Performance of disease severity estimation models of different canopy levels.

Canopy level	Year	Trial	Training		Testing		Lin’s CCC	Coeff.	Intercept
			Precision	RMSE (%)	Precision	RMSE (%)			
Lower	2020	Combined^a^	0.62	8.11	0.56	8.71	0.75	0.99	0.06
	2021	Tar 2	0.93	9.28	0.92	10.42	0.96	1.00	-0.08
		Tar 3	0.94	9.96	0.92	11.39	0.96	1.00	-0.07
Middle	2020	Combined	0.80	9.09	0.78	9.47	0.89	1.00	-0.04
	2021	Tar 2	0.88	9.86	0.87	10.11	0.94	1.00	-0.09
		Tar 3	0.95	7.45	0.93	8.49	0.97	1.00	-0.01
Upper	2020	Combined	0.80	4.97	0.78	5.20	0.88	1.00	-0.02
	2021	Tar 2	0.78	11.05	0.73	11.89	0.86	1.02	-0.14
		Tar 3	0.95	7.23	0.93	8.21	0.97	1.00	-0.03

a Data from four trials in 2020 were combined; RMSE, root mean square error; Lin’s CCC, Lin’s Concordance Correlation Coefficient; Coeff; coefficient for the linear relation between estimated and actual visual assessment.

Estimation models of disease severity were transferred between disease management trials with reasonable performance in 2021; for example, models developed based on data from one trial were used to estimate the visual assessment of the second trial (using data from the second trial). Estimation precision of transferred models (R^2^ = 0.68 to 0.91) and RMSEs (< 14%) were comparable to models trained and tested with data collected from the same trials in 2021. However, agreements between estimated and actual disease severity (Lin’s CCC = 0.78 to 0.92) were slightly lower, while biases for transferred models were higher (intercept = -2.27 to 2.99) compared with counterparts in 2021. Moreover, transferred models using parameters from models of the Tar 2 trial and data from the Tar 3 trial in 2021 underestimated disease severity (coefficient = 1.21 to 1.31, **Figures 2D–F**), while transferred models using parameters from models of the Tar 3 trial and data from the Tar 2 trial overestimated disease severity (coefficient = 0.64 to 0.76). Transferred models using parameters from models of the Tar 3 trial and data from the Tar 2 trial resulted in estimation of disease severity beyond 100%. The model transferability test showed that models can be transferred between disease management trials in 2021, and implied subtle differences between trials. Nevertheless, results indicated that UAS-based multispectral imaging and machine learning can be used to estimate disease severity at different canopy levels or monitor the vertical distribution of tar spot disease on corn and that models can be transferred to disease management trials. However, further improvements are required, including model transferring between years and multispectral cameras.

### 3.2 Monitoring disease progress using data derived from multispectral imaging

**Figure 3:**
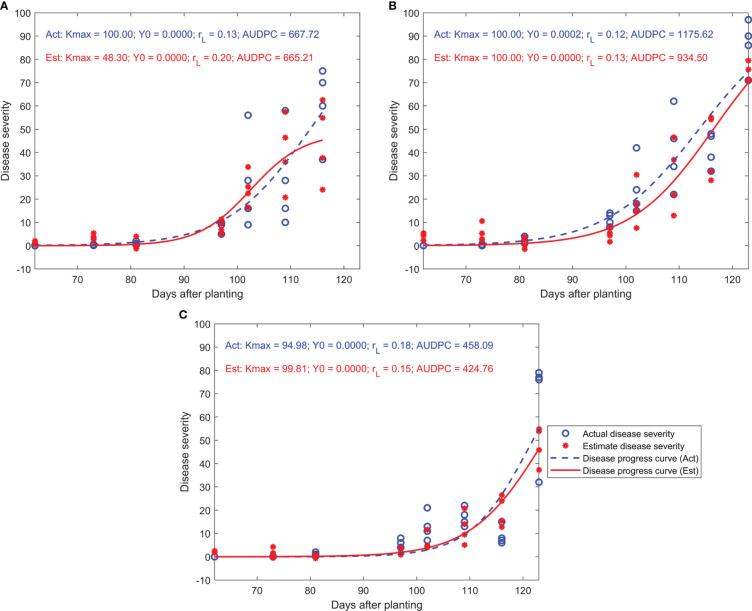
Disease progress curves for actual and estimated disease severity of treatment #7 in the Tar 4 trial during 2020. **(A–C)**: disease progress curves derived from the lower, middle, and upper canopy, respectively; Act, disease progress curve or parameters based on actual disease severity; Est, disease progress curve or parameters based on estimated disease severity; K_max_, the asymptote or the maximum level of disease intensity of a treatment; y_0_, initial disease severity; r_L_, apparent infection rate, AUDPC, area under the disease progress curve.

The shapes of disease progress curves fitted for estimated disease severity were similar to their counterparts fitted for actual (or visual) disease severity in most cases. At the early stages of tar spot infection, curves fitted for actual and estimated disease severity were almost identical (**Figure 3**). However, at the middle or later growth stages, some curves fitted for estimated disease severity diverged from their counterparts, and divergence between fitted curves varied among treatments. The comparison of y_0_ and AUDPC also demonstrated the similarity of disease progress curves derived from actual and estimated disease severity, where no significant difference between y_0_ or AUDPC derived from actual and estimated disease severity was observed in both years and all canopy levels (**Table 4**). However, significant differences (α = 0.05) between K_max_ or r_L_ derived from actual and estimated disease severity were observed, mainly in 2020.

**Table 4:** Comparison of parameters of disease progress curves derived from actual and estimated disease severity using a t-test.

Year	Canopy level	K_max_	Y_0_	r_L_	AUDPC
	Lower	**<.0001**	0.45	**0.00**	0.25
2020	Middle	**0.00**	0.11	**0.00**	0.70
	Upper	0.06	0.21	**0.02**	0.76
	Lower	0.66	0.33	0.28	0.54
2021	Middle	0.13	0.35	0.85	0.80
	Upper	**0.03**	0.48	0.19	0.50

K_max_, the asymptote or the maximum level of disease intensity of a treatment; y_0_, initial disease severity; r_L_, apparent infection rate; AUDPC, area under the disease progress curve; significance level or α of 0.05; number in bold indicates significant difference between parameters derived from actual and estimated disease severity.

### 3.3 Digital phenotyping technologies for disease monitoring

The threshold to determine tar spot onset in a trial through digital phenotyping was selected as 5% of micro-plots (with estimated disease severity over zero), based on evaluation of the number of false warnings obtained by different thresholds. Digital phenotyping can detect the tar spot onset when visual assessment started, even the disease severity was at very low level (<1%) (**Table 5**). Tar spot onset was correctly detected for all combinations of trials and DAP of the lower canopy and most combinations of the middle and upper canopies, with three false warmings for the middle and upper canopies. The estimated incidence rate for each canopy level per trial fluctuated from one DAP to another (e.g., 58%, 29%, 65%, and 90% for lower canopy of Tar 3 in 2020), which was different from the increasing actual incidence rate. Such fluctuation of estimated incidence rate may be due to very low disease severity and minor symptoms in most micro-plots. However, as tar spot intensity increased, the sensitivity of disease onset detection was over 50% in most cases (range of 27% – 98%) at 14 or more days after the practical level of visual detection. Nevertheless, detection of tar spot onset through UAS-based imaging provides a desired level of efficiency and accuracy of estimating incidence rate.

**Table 5:** Summary of detection of tar spot incidence through UAS-based multispectral imaging.

Year	Trial	DAP	Lower canopy				Middle canopy				Upper canopy			
			Max sev. (%)	IR (%)	Est. IR (%)	Sen. (%)	Max sev. (%)	IR (%)	Est. IR (%)	Sen. (%)	Max sev. (%)	IR (%)	Est. IR (%)	Sen. (%)
2020	Tar 4	60	0.30	93	35	35	0.02	20	53	50	0.01	3	55	100
		71	0.50	100	53	53	0.20	90	58	56	0.01	45	33	28
		74	0.70	100	90	90	0.20	95	80	79	0.05	85	35	41
		95	10.00	100	73	73	7.00	100	73	73	4.00	100	83	83
	Tar 3	59	0.10	3	58	100	0.10	6	42	50	0.10	1	51	0
		70	3.00	93	29	31	1.00	94	26	26	0.20	82	32	34
		78	3.00	100	65	65	1.00	100	79	79	0.30	88	68	70
		94	20.00	100	90	90	15.00	100	71	71	6.00	92	69	71
	Tar 1	59	0.30	100	65	65	0.10	33	33	23	0.00	0	**38**	NA
		70	0.30	100	40	40	0.10	90	30	28	0.01	13	43	20
		78	2.00	100	63	63	0.10	98	63	64	0.10	33	58	69
		94	8.00	100	83	83	5.00	100	58	58	4.00	100	65	65
	Tar 2	62	1.00	94	50	47	0.10	17	52	63	0.10	4	71	100
		73	1.00	100	71	71	0.50	100	85	85	0.10	65	73	61
		81	2.00	100	52	52	4.00	100	27	27	2.00	94	46	44
		97	10.00	100	81	81	14.00	100	81	81	8.00	100	98	98
2021	Tar 2	70	0.60	100	56	56	0.00	0	**58**	NA	0.02	33	50	50
		79	2.00	100	48	48	0.10	100	31	31	0.04	42	40	35
		83	8.40	100	69	69	1.80	100	88	88	0.20	73	40	51
		91	20.20	100	88	88	6.00	100	63	63	1.20	100	79	79
		97	8.60	100	94	94	5.40	100	90	90	3.60	100	65	65
	Tar 3	69	0.42	89	37	38	0.34	65	37	40	0.00	0	**57**	NA
		82	1.80	96	63	62	1.10	88	79	81	1.00	64	47	48
		96	6.80	99	60	61	4.00	96	64	64	3.20	93	69	70

DAP, day after planting; Max sev., maximum actual disease severity observed; IR, incidence rate; Est. IR, estimated incidence rate; Sen., sensitivity of detection; NA, not available (no disease was observed); number in bold indicates false negative or false positive cases.

**Figure 4:**
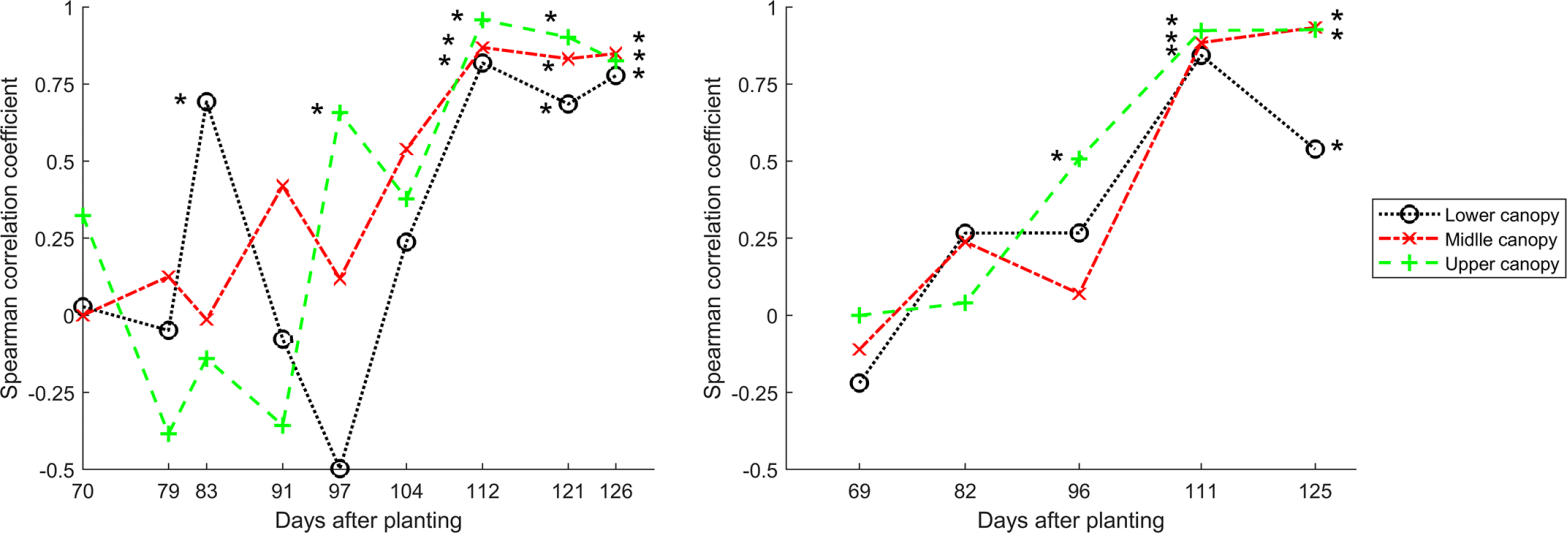
Spearman’s rank correlations between treatment rankings assessed by digital phenotyping and visual assessment for the Tar 2 and Tar 3 trials of 2021. *Indicates significant correlation at a significance level (α) of 0.05.

Significant correlations between treatment rankings assessed by digital phenotyping and visual assessment were observed as early as 71 DAP (**Figure 4**; **Table 6**). However, non-significant correlations were observed between 71 and 100/104 DAP in some trials and canopy levels. One reason for such variation may be because fungicide treatments were applied until about 100 DAP, and the combined effects of fungicides and other factors (e.g., tillage and resistance) may be delayed. However, after 100/104 DAP, significant correlations (*r*_s_ = 0.54 – 0.97) between treatment rankings were found in most cases until the end of the growing season (when senescent leaves were not visually evaluated). In other words, treatment efficacy can be evaluated by UAS-based multispectral imaging near the end of the growing season.

**Table 6:** Spearman’s rank correlations between treatment rankings assessed by digital phenotyping and visual assessment for trials of 2020.

Trial	Canopy level	Parameter				DAP^a^				
			59–62	70–73	78–81	94–97	99–102	106–109	113–116	120–123
Tar 4	Lower	*r*_s_	-0.24	0.23	0.13	0.70	0.01	0.78	NA	NA
		P value	0.51	0.52	0.73	0.03	0.97	0.01	NA	NA
	Middle	*r*_s_	-0.48	0.09	0.04	0.60	0.35	0.96	0.84	0.87
		P value	0.16	0.81	0.92	0.07	0.33	0.00	0.00	0.00
	Upper	*r*_s_	0.29	0.00	0.30	0.32	0.21	0.94	0.78	0.93
		P value	0.42	1.00	0.39	0.37	0.57	0.00	0.01	0.00
Tar 3	Lower	*r*_s_	0.03	0.40	0.19	0.36	0.81	0.95	NA	NA
		P value	0.89	0.10	0.46	0.14	0.00	0.00	NA	NA
	Middle	*r*_s_	-0.08	0.56	0.37	0.43	0.82	0.92	0.97	0.95
		P value	0.76	0.02	0.13	0.07	0.00	0.00	0.00	0.00
	Upper	*r*_s_	-0.26	0.21	0.54	0.48	0.71	0.94	0.96	0.94
		P value	0.30	0.40	0.02	0.04	0.00	0.00	0.00	0.00
Tar 1	Lower	*r*_s_	-0.18	-0.78	0.06	0.54	0.21	0.48	NA	NA
		P value	0.61	0.01	0.87	0.11	0.56	0.16	NA	NA
	Middle	*r*_s_	0.47	0.07	0.47	0.59	0.50	0.79	0.77	0.94
		P value	0.17	0.85	0.17	0.08	0.14	0.01	0.01	0.00
	Upper	*r*_s_	NA	0.18	0.17	0.55	0.43	0.71	0.71	0.86
		P value	NA	0.62	0.64	0.10	0.22	0.03	0.02	0.00
Tar 2	Lower	*r*_s_	-0.29	0.27	0.35	0.85	0.94	0.91	0.81	NA
		P value	0.37	0.41	0.26	0.00	0.00	0.00	0.00	NA
	Middle	*r*_s_	0.04	0.17	0.63	0.40	0.94	0.93	0.81	0.80
		P value	0.90	0.60	0.03	0.19	0.00	0.00	0.00	0.00
	Upper	*r*_s_	-0.34	-0.40	0.43	0.61	0.87	0.81	0.73	0.65
		P value	0.27	0.20	0.16	0.03	0.00	0.00	0.01	0.02

a DAP, day after planting that varied among trials; r_s_, Spearman’s rank correlation coefficient; NA, not available as no disease symptom was observed (upper canopy) or leaves senesced (lower canopy).

## 4 Discussion

Tar spot often develops from the lower canopy in fields with an infection history, which poses a challenge for UAS-based disease monitoring due to occlusion. The results from this study illustrated that it is possible to monitor tar spot epidemics even if they started from the lower canopy and characterize the vertical (different canopy levels) and temporal development of the disease using UAS-based remote sensing and machine learning. Such information is vital for decision-making in fields where corn is grown in micro-plots for research purposes (i.e., hybrid performance and fungicide efficacy trials). This is the unique contribution of our study to UAS-based disease monitoring. The novel methods developed in this study pave the road for UAS-based scouting of tar spot and cereal diseases with similar epidemiological characteristics, such as gray leaf spot, northern corn leaf blight (Stromberg, 2009; Shi et al., 2017) and wheat blast (Gongora-Canul et al., 2020). More importantly, digital phenotyping technologies can generate useful epidemiological information, at the vertical and temporal levels. For instance, derived epidemiological parameters such as disease onset, apparent infection rate, and AUDPC, could potentially be extracted to study disease management tactics and prepare plans to manage tar spot.

The y_0_ or AUDPC derived from actual and estimated disease severity were similar, but significant differences between K_max_ or r_L_ from actual and estimated disease severity were found. Major differences between K_max_ derived from the actual and estimated disease severity were associated with smaller visual values of maximum disease severities. The modeled disease progress curves had a tendency to result in incorrect values of Kmax, when the maximum disease severity of actual or estimated values were in the range of 20 – 30% than when they were in the range of 80-90%. For example, the percentages of micro-plots where the maximum values of actual disease severity did not exceed 50% were 46%, 78%, and 84% of the total number of micro-plots from the lower, middle, and upper canopy levels during 2020. This may be one probable explanation for significant differences between K_max_ during 2020. In general, it was possible to model the temporal development of disease with disease onset or AUDPC data derived from multispectral imaging within the range of available data; however, the modeling of K_max_ and r_L_ requires further improvement.

Although UAS-based multispectral imaging can complement visual assessment when acquiring disease severity information with a large aerial coverage, further research is required to improve performance of detection and quantification of tar spot intensity and enhance the scalability, transferability, and cost effectiveness of UAS-based plant disease survey. Although it is possible to detect tar spot onset through UAS-based imaging, sensitivity of detection of onset was low until 14 or more days after the practical level of visual detection. This may result in false warning (e.g., false positive of tar spot onset) when the disease was in the incubation period showing no symptoms. To increase the accuracy of onset detection through UAS-based imaging, validation of disease onset with a consecutive imaging (e.g., within 3 – 5 days) or integrating visual and/or environmental data with UAS-based data (or data fusion that will be discussed later) are a few recommended options. Hyperspectral or thermal imaging may also be tested for such purposes, as these imagers provide information from different spectra.

Removal of corn tassels from acquired images is a potential way to improve the performance and transferability of the proposed models. The spectral reflectance of corn tassels is different from that of corn leaves (Viña et al., 2004; Shao et al., 2022). Shao et al. reported that tassels lowered the canopy reflectance of the green region, and the impact of tassel reflectance on different vegetation indices varied between growth stages and corn varieties (Shao et al., 2022). The influence of tassel reflectance on canopy reflectance may mask the influence of tar spot on the canopy reflectance. This may make the detection of tar spot more challenging, especially at the early stage of infection when the influence of the disease may be minor. The fact that models from the Tar 3 trial (with only 1 hybrid) showed better performance than those from the Tar 2 trial (with 3 hybrids) in 2021 agrees with this hypothesis. In addition, variation of tassel reflectance introduced by corn varieties may also reduce the transferability of disease severity estimation models derived from different trials. This may be the reason for the lower performance of transferred models compared with those using original data (3 hybrids in the Tar 2 trial while 1 hybrid in the Tar 3 trial in 2021). Previous studies have successfully detected tassels in high-resolution RGB images that were collected by proximal and UAS-based aerial images at relatively low altitudes (<= 20 m) (Lu et al., 2017; Liu et al., 2020; Zan et al., 2020). However, it is challenging to detect and remove tassels in low-resolution multispectral images collected at higher altitudes (e.g., ground sampling distance of 1.5 and 3.5 cm for images collected at 30 and 50 m, respectively, in this study). Future research on removing tassels from acquired images and their impact on canopy reflectance in disease monitoring is desired.

Data fusion is another potential way to improve the performance and scalability of disease estimation models in monitoring tar spot, where environmental data and UAS-based multispectral images are integrated and used as inputs during model development. Data fusion has been used to monitor diseases, and better performance has been reported for methods using fused data (Zhang et al., 2014; Yun et al., 2015; Yuan et al., 2017; Zheng et al., 2021). For example, Zheng et al. monitored the occurrence of wheat yellow rust disease using meteorological data and multispectral images collected by Sentinel-2 satellite (2021). Models using fused data (accuracy of 68.4% – 84.2%) increased the classification accuracy by 5.2% to 10.5%, compared with those using only multispectral images. Currently, the performance of our tar spot detection and quantification methods was susceptible to degradation under abiotic stresses (e.g., nutrient deficiency) and other biotic stresses (e.g., diseases such as gray leaf spot and northern corn leaf blight). Integrating data from two primary components of the disease triangle, i.e., environment (e.g., air temperature, humidity, and leaf wetness) and host (multispectral reflectance of plants), may reduce the uncertainty introduced by other biotic or abiotic stresses. For example, environmental conditions favorable for the occurrence of tar spot (17 – 22°C, relative humidity (RH) > 75%, and more than seven hours of leaf wetness per night) are different from these for northern corn leaf blight (18 – 27°C and six to 18 hours of leaf wetness) and gray leaf spot (22 – 30°C and nightly RH > 90%) (Hock et al., 1995; Paul, 2003; Wise, 2011). The integration of environmental data in our disease monitoring models may reduce the uncertainty or false warnings.

## 5 Conclusion

Tar spot, a threat to corn production, is a disease that regularly appears at the lower canopy of plants in fields with a history of tar spot infections. This study evaluated digital phenotyping technologies and machine learning approaches for monitoring tar spot epidemics at the different canopy and temporal levels. The results showed that models developed with UAS-based multispectral imaging and machine learning could estimate disease severity for the upper, middle and lower canopy levels exhibiting encouraging performance. The estimated precision or R^2^ of models ranged from 0.75 to 0.93 in most cases, while the concordance estimated using Lin’s CCC varied between 0.75 and 0.97, showing moderate to high agreements between estimated and actual disease severity. In addition, the developed disease estimation models were effectively transferred between disease management trials. The temporal development of tar spot disease was modeled using data derived from multispectral images, showing no significant difference between y_0_ or AUDPC derived from actual and estimated disease severity. Successful results were obtained when applying information derived from digital phenotyping technologies to disease monitoring, for example, detection of tar spot onset and evaluation of the efficacy of disease management tactics. Epidemiological information derived, such as disease severity from different canopy and temporal levels, disease onset, and efficacy of disease management tactics need to be explored in larger field areas and evaluated for their value in supporting decision-making. In addition, methods developed in this study might be applied to other diseases with similar epidemiological characteristics, but additional research is needed. Further studies are required to improve the performance in detection and quantification of tar spot intensity and enhance the scalability and transferability of our methods to different regions and corn varieties. Removal of corn tassels from acquired images and fusion of aerial multispectral images with environmental data are future work that may lead to better performance of our methods.

## Data availability statement

The original contributions presented in the study are included in the article/supplementary material, further inquiries can be directed to the corresponding author/s.

## Author contributions

CZ and CC conceived the conceptualization of this study. CC, DT, SG, and SS provided resources and fundings. DT, TR, and CD-S implemented the field works. CC, BL, MF-C, AC-S, D-YL, CG-C, TR, CD-S, SO acquired and curated the data. CZ, CG-C, and CC analyzed the data. CZ and CC prepared for the first draft of the manuscript. All authors contributed to the article and approved the submitted version.
