# Supplementary material for: Monitoring tar spot disease in corn at different canopy and temporal levels using aerial multispectral imaging and machine learning
Source: Front Plant Sci. 2023 Jan 23;13:1077403. doi: 10.3389/fpls.2022.1077403 (PMC9900023; doi:10.3389/fpls.2022.1077403)
Supplement: Supplementary file 1 [file DataSheet_1.docx]

**Monitoring tar spot disease in corn at different canopy and temporal levels using aerial multispectral imaging and machine learning**

Chongyuan Zhang^1^, Brenden Lane^1^, Mariela Fernandez-Campos^1^, Andres Cruz-Sancan^1^, Da-Young Lee^1^, Carlos Gongora-Canul^1^, Tiffanna J. Ross^1^, Camila R. Da Silva^1^, Darcy E. P. Telenko^1^, Stephen B. Goodwin^2^, Steven R Scofield^2^, Sungchan Oh**^3^**, Jinha Jung**^4^**, and Christian D. Cruz^1^

^1^ Department of Botany and Plant Pathology, Purdue University, West Lafayette, Indiana 47907, U.S.A.

^2^ USDA-Agricultural Research Service, Crop Production and Pest Control Research Unit, West Lafayette, Indiana 47907, U.S.A.

^3^ Institute for Plant Sciences, Purdue University, West Lafayette, Indiana 47907, U.S.A.

^4^ Lyles School of Civil Engineering, Purdue University, West Lafayette, Indiana 47907, U.S.A.***** Correspondence: C. D. Cruz, [cruz113@purdue.edu](mailto:cruz113@purdue.edu)


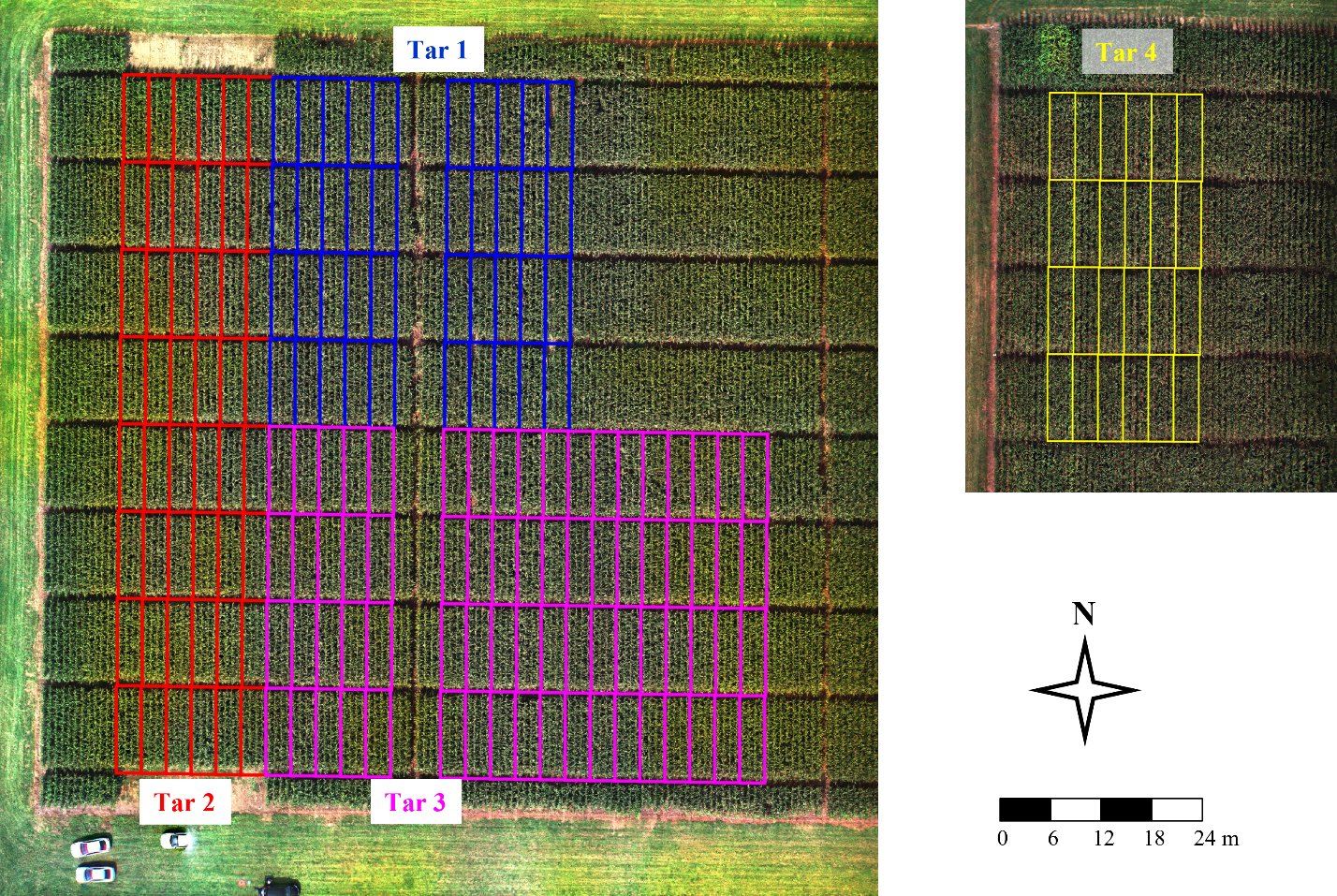


**Supplementary Figure 1.** Plot layouts of tar spot experiments during the 2020 growing season.


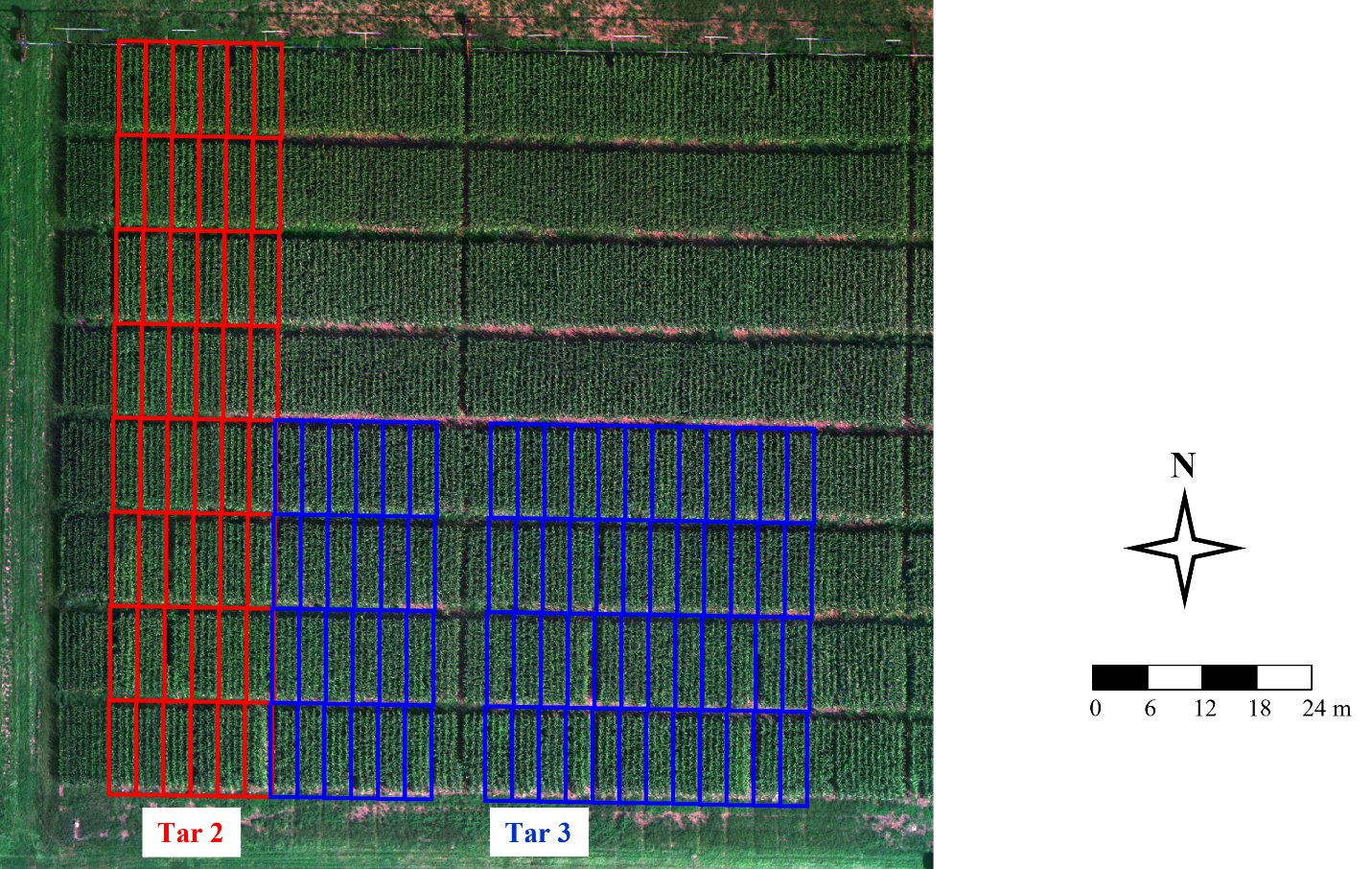


**Supplementary Figure 2.** Plot layouts of tar spot experiments during the 2021 growing season.


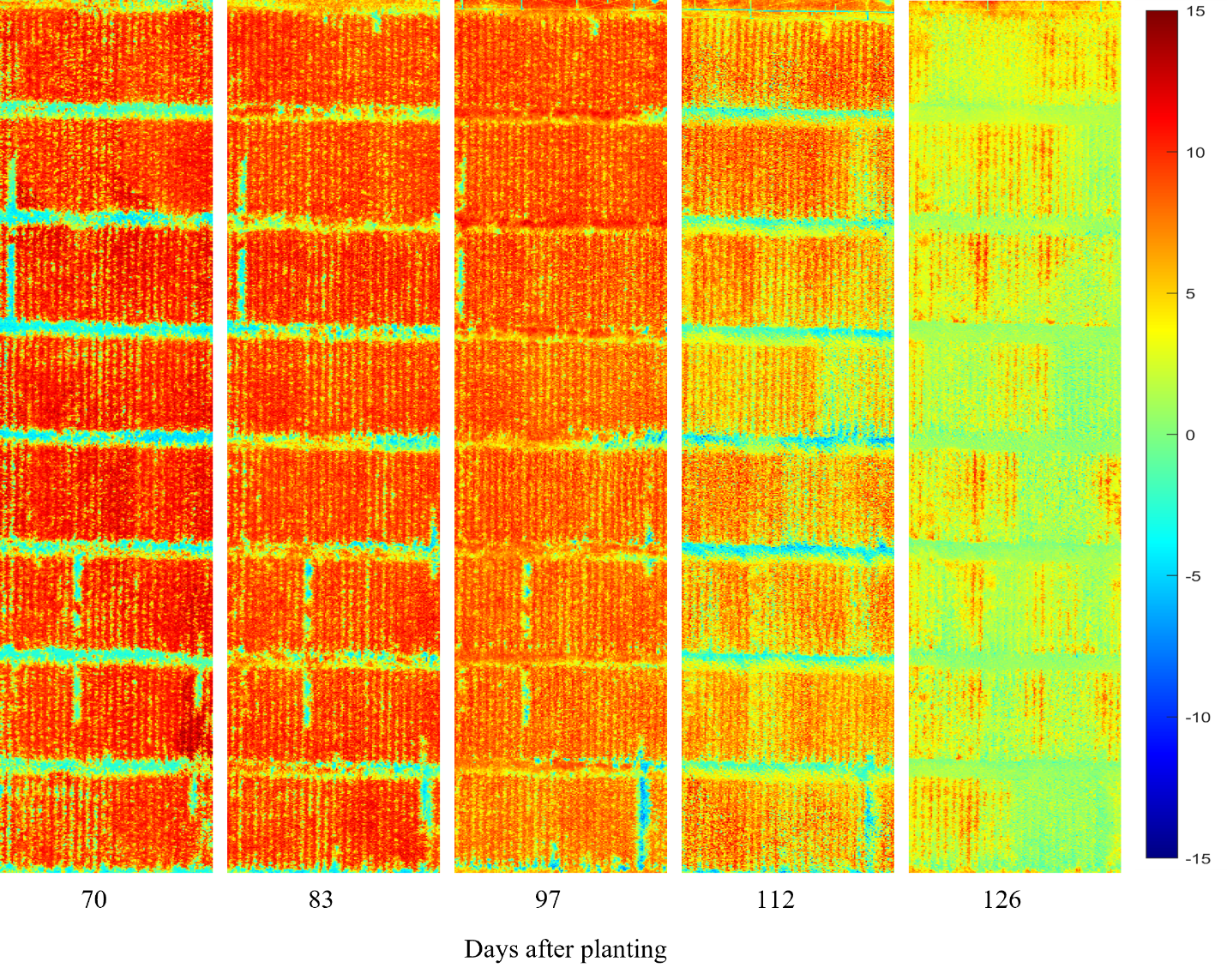


**Supplementary Figure 3.** Renormalized difference vegetation index (RDVI) map for plots (every four rows) from different days after planting of Tar 2 trial of 2021.


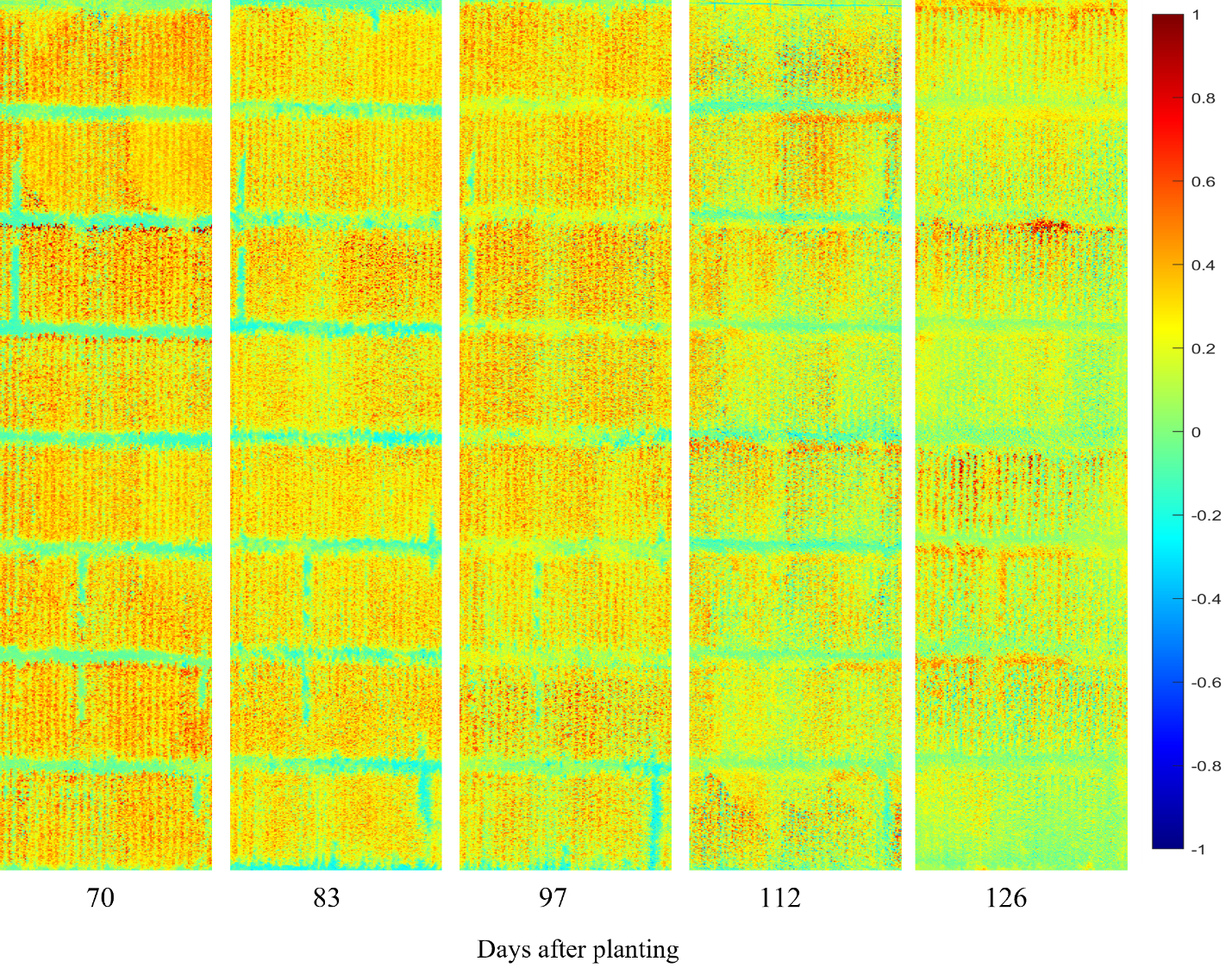
**Supplementary Figure 4.**  Red edge normalized difference vegetation index (NDRE) map for plots (every four rows) from different days after planting of Tar 2 trial of 2021.

**Supplementary Table 1.** Vegetation indices calculated using multispectral images in this study.

| Vegetation index | Full name | Equation^a^ |
| --- | --- | --- |
| NDVI | Normalized difference vegetation index | ${(NIR-Red)}/{(NIR+Red)}$ |
| GNDVI | Green normalized difference vegetation index | ${(NIR-Green)}/{(NIR+Green)}$ |
| SAVI | Soil adjusted vegetation index | ${1.5*(NIR-Red)}/{(NIR+Red+0.5)}$ |
| NDRE | Red edge normalized difference vegetation index | ${(NIR-RE)}/{(NIR+RE)}$ |
| TVI | Triangular vegetation index | 0.5 * (120 * (NIR – Green) – 200*(Red – Green)) |
| ExG | Excess green | 2 * Green – Red – Blue |
| SR | simple ratio | $\mathrm{NIR}/\mathrm{Red}$ |
| PSRI | plant senescence reflectance index | ${(Red-Green)}/\mathrm{NIR}$ |
| G | green index | $\mathrm{Green}/\mathrm{Red}$^b^ |
| MCARI2 | modified chlorophyll absorption in reflectance index | $\frac{1.5*(2.5*(NIR-Red)-1.3*(NIR-Green))}{\sqrt{{(2*NIR+1)}^{2}-(6*NIR-5*\sqrt{Red})-0.5}}$ |
| GRVI | green-red vegetation index | ${(Green-Red)}/{(Green+Red)}$ |
| RDVI | renormalized difference vegetation index | ${(NIR-Red)}/\sqrt{(NIR+Red)}$ |

^a^ (Harris Geospatial Solutions, 2022), equations can be found in the referred website, except for the G; ^b^ (Zarco-Tejada et al., 2005); NIR: near infrared band; RE: red edge band.
